# Shear wave elastography: A noninvasive approach for assessing acute kidney injury in critically ill patients

**DOI:** 10.1371/journal.pone.0296411

**Published:** 2024-01-11

**Authors:** Banghong Qiang, Qiancheng Xu, Youjun Pan, Junli Wang, Chunyun Shen, Xiaozhuang Peng, Wenwen Shen, Yu Zhang, Xiangming Zhu

**Affiliations:** 1 Anhui Medical University, Hefei, Anhui, China; 2 Department of Ultrasound Medicine, The First Affiliated Hospital of Wannan Medical College (Yijishan Hospital of Wannan Medical College), Wuhu, Anhui, China; 3 Department of Ultrasound Medicine, Wuhu Hospital, East China Normal University (The Second People’s Hospital, Wuhu), Wuhu, Anhui, China; 4 Department of Critical Care Medicine, The First Affiliated Hospital of Wannan Medical College (Yijishan Hospital of Wannan Medical College), Wuhu, Anhui, China; 5 Department of Critical Care Medicine, Wuhu Hospital, East China Normal University (The Second People’s Hospital, Wuhu), Wuhu, Anhui, China; Istituto Di Ricerche Farmacologiche Mario Negri, ITALY

## Abstract

Traditional markers, such as serum creatinine and blood urea nitrogen, frequently show delayed elevations following acute kidney injury (AKI), limiting their utility for prompt detection and timely intervention in AKI management. Shear wave elastography (SWE) exhibits potential for AKI diagnosis by measuring tissue stiffness. Our study aimed to evaluate the diagnostic performance of SWE in detecting AKI by measuring the stiffness of kidney tissue. Between July 2022 and December 2022, a total of 103 consecutive participants who met the eligibility criteria were prospectively enrolled, underwent SWE measurements, and were classified into AKI or non-AKI groups based on the 2012 Kidney Disease: Improving Global Outcomes (KDIGO) criteria. A receiver operating characteristic (ROC) curve was drawn to examine the feasibility of differentiating between AKI and non-AKI patients and assessing diagnostic performance. The effects of tissue anisotropy on SWE measurements were also examined. Our results revealed that patients in the AKI group exhibited significantly increased stiffness values in specific kidney regions compared with those in the non-AKI group. For the diagnosis of AKI, the optimal cut-off values were identified as 9.9 kPa, 2.9 kPa, and 4.4 kPa for the upper pole medulla, middle cortex, and middle medulla, respectively, in the longitudinal plane. Correspondingly, the areas under the ROC curves for these regions were 0.737 (95% confidence interval [CI]: 0.637, 0.822), 0.736 (95% CI: 0.637, 0.821), and 0.784 (95% CI: 0.688, 0.861). Additionally, we observed a significant variability in stiffness values due to tissue anisotropy, specifically in the segments of the upper pole cortex, and medulla across both longitudinal and transverse planes. SWE serves as a noninvasive approach for the quantification of tissue stiffness and shows promise as an adjunctive tool for the assessment of AKI.

## Introduction

Approximately 10 to 15% of hospitalized patients in the United States suffer from acute kidney injury (AKI) [1], which is characterized by a rapid increase in serum creatinine (sCr) and/or a dramatic reduction in urine output. A growing ageing population has led to an increase in its incidence in intensive care units (ICUs) worldwide over the past decades [2]. Of note, more than half of critically ill patients develop AKI [3]. Furthermore, AKI has been significantly correlated with mortality and morbidity in ICUs patients [4–6].

Currently, AKI is diagnosed based on the 2012 Kidney Disease Improving Global Outcomes (KDIGO) guidelines [7]. Central to the diagnostic protocol for AKI is the assessment of a decline in glomerular filtration rate (GFR), which serves as a key and integrative indicator of kidney function. However, the direct measurement of GFR in clinical practice presents inherent challenges. Hence, the sCr level is used to estimate the GFR and thereby facilitate the timely diagnosis and management of AKI. Studies have shown that a mild elevation of sCr contributes to adverse AKI outcomes [8,9]. Additionally, urine volume is regarded as a sensitive indicator of renal function and tubular injury [10,11]. However, the relationship among urine volume, the GFR and renal tubular damage is intricate, and changes in these indicators may be delayed, subtle, and nonspecific. These disadvantages restrict the use of these factors in the early diagnosis of AKI. Therefore, exploration of a noninvasive and reproducible alternative method to detect early changes in AKI is crucial for holistic management of patients with AKI.

Shear wave elastography (SWE) can quantify tissue stiffness [12] and serves probably as a noninvasive approach for diagnosing and monitoring AKI. SWE possesses high specificity and sensitivity for distinguishing between normal and cirrhotic livers [13,14]. The kidney is a highly compartmentalized and heterogeneous organ, SWE has been used to evaluate kidney stiffness in the native kidney [15,16], with demonstrated accuracy and reproducibility. Previous studies have shown that stiffness values vary with tissue anisotropy in the kidney in experimental animal models [17,18] and kidney allograft [19]. However, to our knowledge, few studies have applied SWE as a noninvasive approach to quantify the stiffness of kidney tissue for detecting AKI. Moreover, SWE measurements on the effects of tissue anisotropy in different segments and compartments of the kidney in the longitudinal and transverse planes have also never been evaluated.

The aim of this study was to assess the diagnostic performance of SWE for AKI in critically ill patients. Additionally, the study sought to scrutinize the effect of tissue anisotropy on SWE measurements and to assess the reliability of these measurements.

## Materials and methods

### Participants

Ethical approval for this prospective, single-center study (clinical trial number ChiCTR2200062133) was secured from the Institutional Review Board of The Second People’s Hospital, Wuhu (approval number: 2021–12). This study was performed in accordance with the Declaration of Helsinki and Declaration of Istanbul. All participants, or their authorized representatives, provided written informed consent. The study did not harm or infringe upon the rights of healthy volunteers or participants. The participants’ demographic and laboratory data were also collected.

From July 2022 to December 2022, participants presenting with clinical findings suggestive of AKI and non-AKI were prospectively enrolled based on the 2012 KDIGO guidelines. Upon admission, all participants underwent a comprehensive clinical evaluation, assessment of laboratory parameters, and SWE examination within 24 hours of their admission. Participants were considered to have AKI if they met any of the following criteria: an increase in sCr of ≥ 0.3 mg/dl (≥ 26.5 μmol/l) within 48 hours; an increase in sCr to ≥ 1.5 times the baseline value within the previous 7 days; or urine volume ≤0.5 ml/kg/h for 6 hours. The inclusion criteria for the study were as follows: (a) acute physiology and chronic health evaluation II (APACHE II) ≥15 and (b) signed informed consent. The exclusion criteria were as follows: (a) age younger than 18 years old, (b) pregnant women, (c) previous history of CKD, (d) AKI caused by retrorenal obstruction, (e) history of renal allograft, (f) body mass index (BMI)>35 kg/m^2^, and (g) poor corticomedullary differentiation on conventional B-mode US.

To examine the inter- and intraobserver reliabilities of SWE measurements, we prospectively recruited a cohort comprised of both healthy volunteers and critically ill patients. Concurrently, to evaluate the effect of tissue anisotropy, a cohort comprised solely of critically ill patients was enrolled. The details of the inclusion and exclusion criteria and SWE measurements are provided in **Supporting information.**

All participants’ demographic and medical details were collected by reviewing electronic medical records or interviews.

### Imaging protocol

Conventional B-mode US and SWE were performed with a curved 5C1 broadband transducer (frequency range: 1.0–5.7 MHz) on an ACUSON Sequoia ultrasound system (Siemens Medical Solutions, Mountain View, CA, USA). All measurements were performed by one of the two attending sonographers (B.H. and C.Y., with 19 and 20 years of experience in kidney imaging and including 12 years of experience with US elastography, respectively) who were blinded to the participants’ data. Participants were placed in the left decubitus position at a 45-degree angle to provide the shortest distance to the right kidney. Subsequently, a conventional B-mode US was performed, and tissue harmonic imaging was applied to optimize image quality. Following this, the SWE mode in which shear waves were generated in tissue using acoustic radiation force generated by focused ultrasound pulses was activated. The pressure exerted by the probe was reduced as much as possible during imaging to avoid compression of the kidney. A fixed sampling frame was set. After an update, the quality map displayed yellow‒green (no large red areas in the area of interest). The region of interest (ROI) was selected using a standardized diameter of 3×3 mm^2^ and placed within the covered cortex and medulla either parallel or perpendicular to the main axis of the kidney (the longitudinal or transverse planes) in different segments (upper pole, middle, lower pole). The mean stiffness values were obtained from the ROI in the SWE image. SWE measurements were considered valid if the interquartile range (IQR) was < 30% of the median value [20]. A total of 12 SWE measurements from 3 consecutive SWE acquisitions were obtained for each kidney segment and compartment in the longitudinal and transverse planes. Then, the mean of the 12 measurements was documented as Young’s modulus (YM) in kPa for statistical analyses. Kidney imaging and accessible SWE measurements were obtained for the right kidney for all participants.

### Statistical analysis

For continuous variables, data are presented as either means with standard deviations or medians with interquartile ranges according to the type of data distribution (normal or skewed). To verify the normality of a variable’s distribution, the Kolmogorov‒Smirnov test was conducted. Frequencies and percentages were used to describe qualitative variables. The inter- and intraobserver reliability was examined by the intraclass correlation coefficient (ICC). Agreement was categorized into three levels: poor (ICC<0.40), fair to good (ICC = 0.40 to 0.75) or excellent (ICC>0.75) [21]. For different kidney segments and compartments, the stiffness values of the longitudinal and transverse planes were compared using the Mann-Whitney U-test for continuous variables. The area under the ROC curve was compared by a nonparametric test to evaluate the diagnostic performance of SWE. The sensitivity, specificity, positive predictive value, and negative predictive value were calculated for optimal cut-off values. The optimal cut-off values for the detection of AKI were selected to maximize sensitivity and specificity. *p*-values < 0.05 were considered statistically significant. Statistical analyses were performed with SPSS software (version 24.0, IBM Corporation, Armonk, NY, USA) and MedCalc software (version 13, MedCalc Software bvba, Ostend, Belgium).

## Results

### Participants characteristics

**Fig 1:**
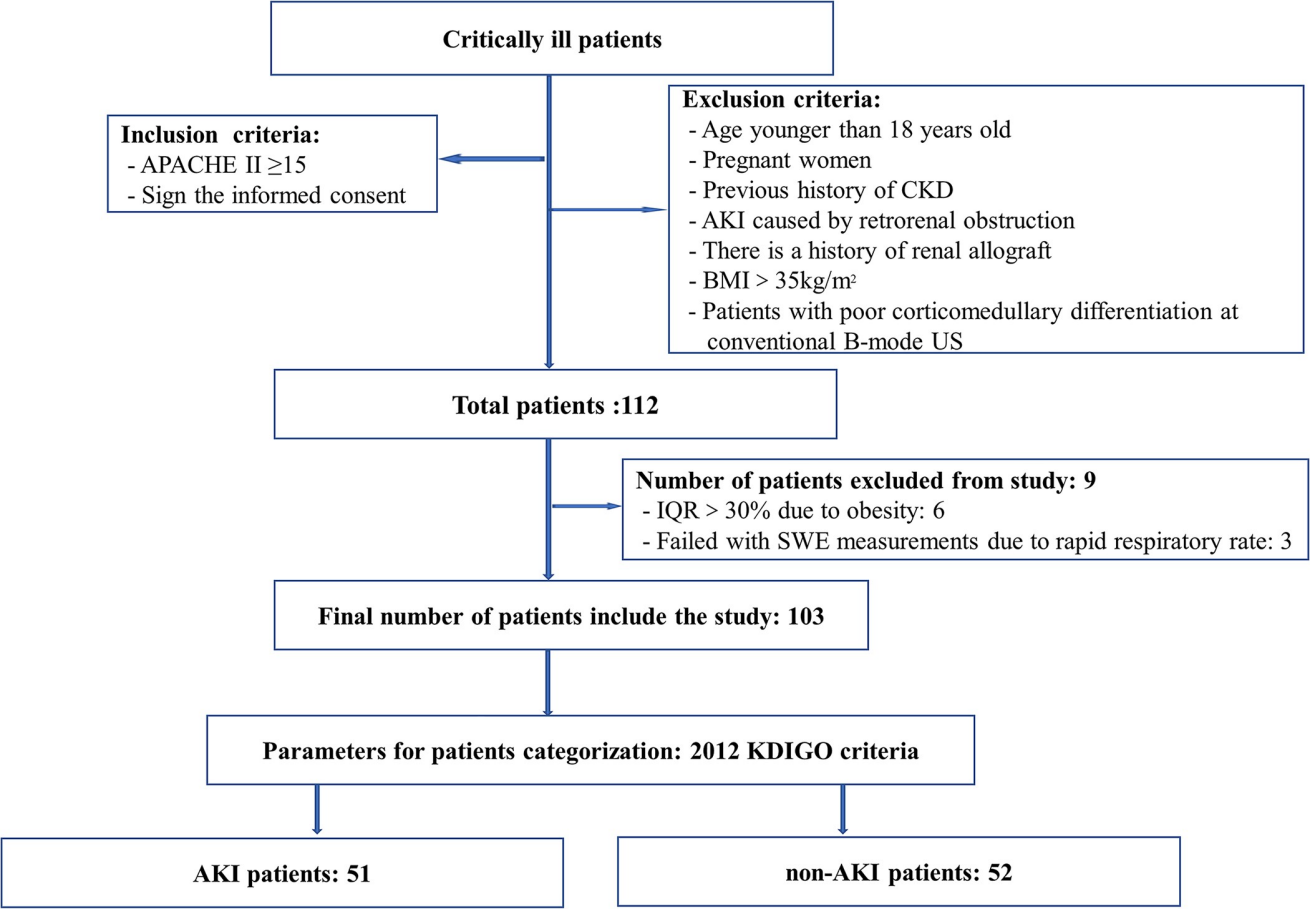
Study flowchart. KDIGO, Kidney Disease: Improving Global Outcomes; SWE, shear wave elastography; APACHE II, acute physiology and chronic health evaluation II.

A total of 112 participants who met the eligibility criteria were prospectively enrolled for SWE measurements. Nine of the participants were excluded, SWE measurements in 6 of these participants showed IQR>30% due to obesity, and an additional 3 participants were excluded due to a rapid respiratory rate. Ultimately, 103 eligible participants met the inclusion criteria. Among them, 51 participants (49.51%) were diagnosed with AKI, with a median age of 71 years (IQR, 62 to 79 years). Additionally, there were 52 participants (50.49%) classified as non-AKI, with a median age of 69 years (IQR, 63 to 74 years). All these participants were enrolled in the study. A flowchart depicting the inclusion and exclusion criteria is presented in **Fig 1**, and the participants’ characteristics are presented in **Table 1**. The sequential organ failure assessment (SOFA) score, heart rate, and 28-day mechanical ventilation time were higher in the AKI group compared with the non-AKI group, whereas the platelet count was lower. The percentage of participants receiving vasoactive medications was significantly higher in the AKI group compared with the non-AKI group. However, no significant difference in 28-day mortality was noted between the two groups.

**Table 1:** Clinical characteristics of participants in the AKI and non-AKI groups.

Characteristic	AKI group (n = 51)	non-AKI group (n = 52)	*p*-value
**Baseline**			
Male (%)	38 (74.5)	36 (69.2)	0.551
Age (years)	71 [62–79]	68.5 [55–78]	0.122
Body mass index (kg/m^2^)	22.49 [21.22–25.69]	23.35 [21.30–25.33]	0.877
APACHE II	19 [15–23]	16 [12–18.75]	0.124
SOFA	7 [5–9]	4.5 [3–6]	<0.001
Mechanical ventilation?	24 (47.1)	24 (46.2)	0.927
Temperature (°C)	36.7 [36.3–37.3]	36.6 [36.4–37]	0.414
Heart rate (bpm)	87 [77–102]	74 [66–89]	0.001
Respiratory rate (bpm)	14 [12–20]	15 [13–18]	0.955
Systolic pressure (mmHg)	118 [107–138]	124 [113.25–144.5]	0.119
Diastolic pressure (mmHg)	71 [59–79]	72 [62.25–79]	0.537
Fraction of inspired oxygen (%)	40 [40–45]	40 [40–45]	0.409
Positive end expiratory pressure (cmH_2_O)	5 [5–7]	5 [5–5]	0.408
Plateau pressure (cmH_2_O)	18 [15.75–21.25]	19.5 [17–21.75]	0.451
Peripheral capillary oxygen saturation (%)	99 [97.75–100]	99.5 [98–100]	0.287
Central venous pressure (cmH_2_O)	10 [7–12]	8 [6.6–10.05]	0.122
**Laboratory tests**			
Urea (mmol/L)	15.35 [8.2–26.3]	5.2 [4.13–7.98]	<0.001
Serum creatinine (μmmol/L)	167 [111–277]	51.5 [41.25–70.25]	<0.001
White blood cell count (×10^9^/L)	10.95 [7.93–14.51]	9.65 [7.10–11.87]	0.303
Platelet count (×10^9^/L)	106 [52–206]	172 [120.5–258.5]	0.001
Procalcitonin (ng/mL)	3.91 [0.86–13.87]	0.24 [0.1–1.13]	<0.001
Lactate (mmol/L)	1.6 [1.03–2.68[	1.35 [0.83–1.98]	0.245
**Comorbidity**			
Hypertension (%)	20 (39.2)	29 (5.8)	0.093
Diabetes (%)	16 (31.4)	4 (7.7)	0.002
Coronary heart disease (%)	14 (27.5)	4 (7.7)	0.008
Chronic pulmonary disease (%)	5 (9.8)	5 (9.6)	0.974
Malignant solid tumor (%)	8 (15.7)	8 (15.4)	0.966
Others (%)	13 (25.5)	17 (32.7)	0.421
**Etiology of admission to ICU**			
Sepsis (%)	32 (62.7)	2 (3.8)	<0.001
Respiratory failure (%)	13 (25.5)	12 (23.1)	0.775
AECOPD (%)	1 (2.0)	4 (7.7)	0.371
Acute left heart failure (%)	3 (5.9)		
Trauma (%)	13 (25.5)	32 (61.5)	<0.001
Others (%)	7 (13.7)	9 (7.3)	0.616
**Treatment**			
Vasoactive drugs	15 (29.4)	4 (7.7)	0.004
CRRT(%)	15 (29.4)		
**KDIGO grade**			
I	28 (54.9)		
II	15 (29.4)		
III	8 (15.7)		
**Outcomes**			
28-day mortality	31 (60.8)	33 (63.5)	0.779
28-day Mechanical ventilation time (days)	8 [4–19.25]	5 [2–11]	0.021
28-day CRRT time (days)	4 [2–6]		
ICU stay time (days)	15 [6.75–24.5]	12 [6–19.75]	0.384
Hospital stay (days)	26.5 [14.75–39.5]	31 [21–45.5]	0.111

Data are presented as median with interquartile range. SOFA, sequential organ failure assessment; APACHE II, acute physiology and chronic health evaluation II; AECOPD, acute exacerbation of chronic obstructive pulmonary disease; ICU, intensive care unit; CRRT, continuous renal replacement therapy.

### Effect of Anisotropy on critically ill patients

A Mann-Whitney U-test was performed to compare different kidney segments and compartments in the longitudinal and transverse planes. The results revealed significant differences in the segments of the upper pole cortex and medulla when observed in both the longitudinal and transverse planes. No significant differences were noted between the longitudinal and transverse planes among the other segments and compartments **(Table 2)**.

**Table 2:** Effect of anisotropy on critically ill patients’ stiffness values by SWE measurements (kPa).

Characteristic			95% CI	*p*-value
**Segments**	**Longitudinal plane**	**Transverse plane**		
Upper pole cortex	9.95 ± 4.81	7.46 ± 3.91	1.598, 3.383	<0.001
Upper pole medulla	9.98 ± 4.65	8.54 ± 4.33	0.513, 2.376	0.012
Middle cortex	5.18 ± 3.98	4.57 ± 2.65	-0.036, 1.259	0.338
Middle medulla	5.07 ± 3.13	4.87 ± 2.94	-0.329, 0.729	0.544
Lower pole cortex	4.44 ± 2.59	3.87 ± 2.03	0.097, 1.046	0.165
Lower pole medulla	4.24 ± 2.40	4.23 ± 2.46	-0.537, 0.556	0.905
**Compartments**	**Cortex**	**Medulla**		
Longitudinal upper pole	9.95 ± 4.81	9.98 ± 4.65	-0.801, 0.624	0.800
Longitudinal middle	5.18 ± 3.98	5.07 ± 3.13	-0.494, 0.766	0.898
Longitudinal lower pole	4.44 ± 2.59	4.24 ± 2.40	-0.229, 0.625	0.571
Transverse upper pole	7.46 ± 3.91	8.54 ± 4.33	-1.778, 0.356	0.061
Transverse middle	4.57 ± 2.65	4.87 ± 2.94	-0.779, 0.187	0.609
Transverse lower pole	3.87 ± 2.03	4.23 ± 2.46	-0.861, 0.132	0.374

Data are presented as median with interquartile range. SWE, shear wave elastography; ICC, intraclass correlation coefficient; 95% CI, 95% confidence interval.

### Predominant characteristics and comparison of stiffness values in the AKI and non-AKI groups

**Fig 2:**
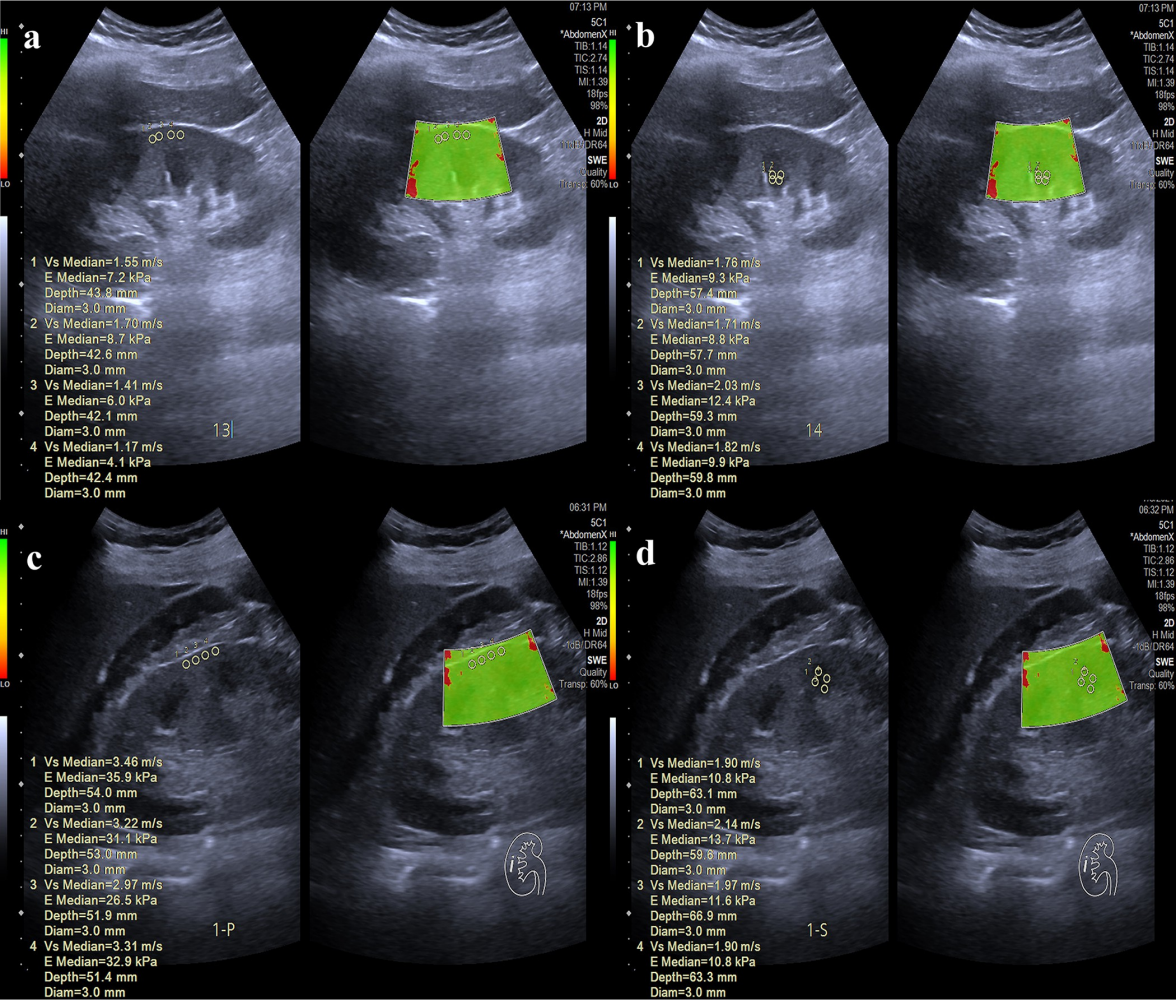
The **s**tiffness values of the middle cortex (**a)** and medulla (**b)** in the longitudinal plane by SWE measurement in a 40-year-old man without AKI. The **s**tiffness values of the middle cortex (**c)** and medulla (**d)** in the longitudinal plane by SWE measurements in a 58-year-old man with AKI. SWE, shear wave elastography; AKI, acute kidney injury.

The SWE images from participants in the AKI and non-AKI groups are presented in **Fig 2**. The resistance index (RI) was higher in the AKI group, whereas the end diastolic velocity (EDV) was lower. A statistically significant difference in kidney tissue stiffness, as determined by SWE measurements, were detected between participants with AKI and those without AKI. These differences were observed in several segments and compartments: longitudinal upper pole cortex, longitudinal upper pole medulla, longitudinal middle cortex, longitudinal middle medulla, transverse upper pole medulla, and transverse middle medulla. However, it is worth noting that no substantial differences were observed in other segments and compartments, which include longitudinal lower pole cortex, longitudinal lower pole medulla, transverse upper pole cortex, transverse middle cortex, transverse lower pole cortex, and transverse lower pole medulla **(Table 3)**.

**Table 3:** Characteristics and stiffness values in the AKI and non-AKI groups by SWE measurements (kPa).

Characteristic	AKI group (n = 51)	non-AKI group (n = 52)	*p*-value
**Baseline**			
The length of kidney	110 [101.45–117.65]	106.75 [100.3–115.53]	0.604
Peak systolic velocity (cm/s)	44.85 [32.53–53.75]	47.30 [36.73–61.83]	0.160
End diastolic velocity (cm/s)	10.85 [7.75–14.73]	14.50 [10.45–20.15]	0.005
RI	0.74 [0.71–0.82]	0.70 [0.65–0.76]	0.003
**Longitudinal plane (kPa)**			
Upper pole cortex	11.35 [7.95–14.70]	8.05 [4.93–11.45]	0.002
Upper pole medulla	11.75 [9.00–15.38]	7.80 [6.00–11.10]	<0.001
Middle cortex	5.00 [4.00–8.00]	3.20 [2.33–5.08]	<0.001
Middle medulla	5.70 [3.80–7.00]	3.20 [2.40–4.40]	<0.001
Lower pole cortex	4.35 [2.63–6.38]	3.60 [2.63–4.93]	0.117
Lower pole medulla	4.00 [2.65–5.63]	3.35 [2.50–5.23]	0.177
**Transverse plane (kPa)**			
Upper pole cortex	8.00 [4.83–10.58]	6.70 [4.00–8.98]	0.058
Upper pole medulla	9.35 [6.93–12.18]	7.00 [5.00–9.40]	0.001
Middle cortex	3.95 [3.03–7.15]	3.80 [2.53–5.18]	0.150
Middle medulla	5.35 [3.65–6.65]	3.35 [2.10–5.58]	0.004
Lower pole cortex	3.75 [2.35–5.70]	3.45 [2.60–4.73]	0.135
Lower pole medulla	3.75 [2.45–5.30]	3.60 [2.10–5.53]	0.612

Data are presented as median with interquartile range or frequencies with percentages. SWE, shear wave elastography; RI, resistance index; ICC, intraclass correlation coefficient; 95% CI, 95% confidence interval.

### Diagnostic performance of SWE in differentiating the AKI and non-AKI groups

**Fig 3:**
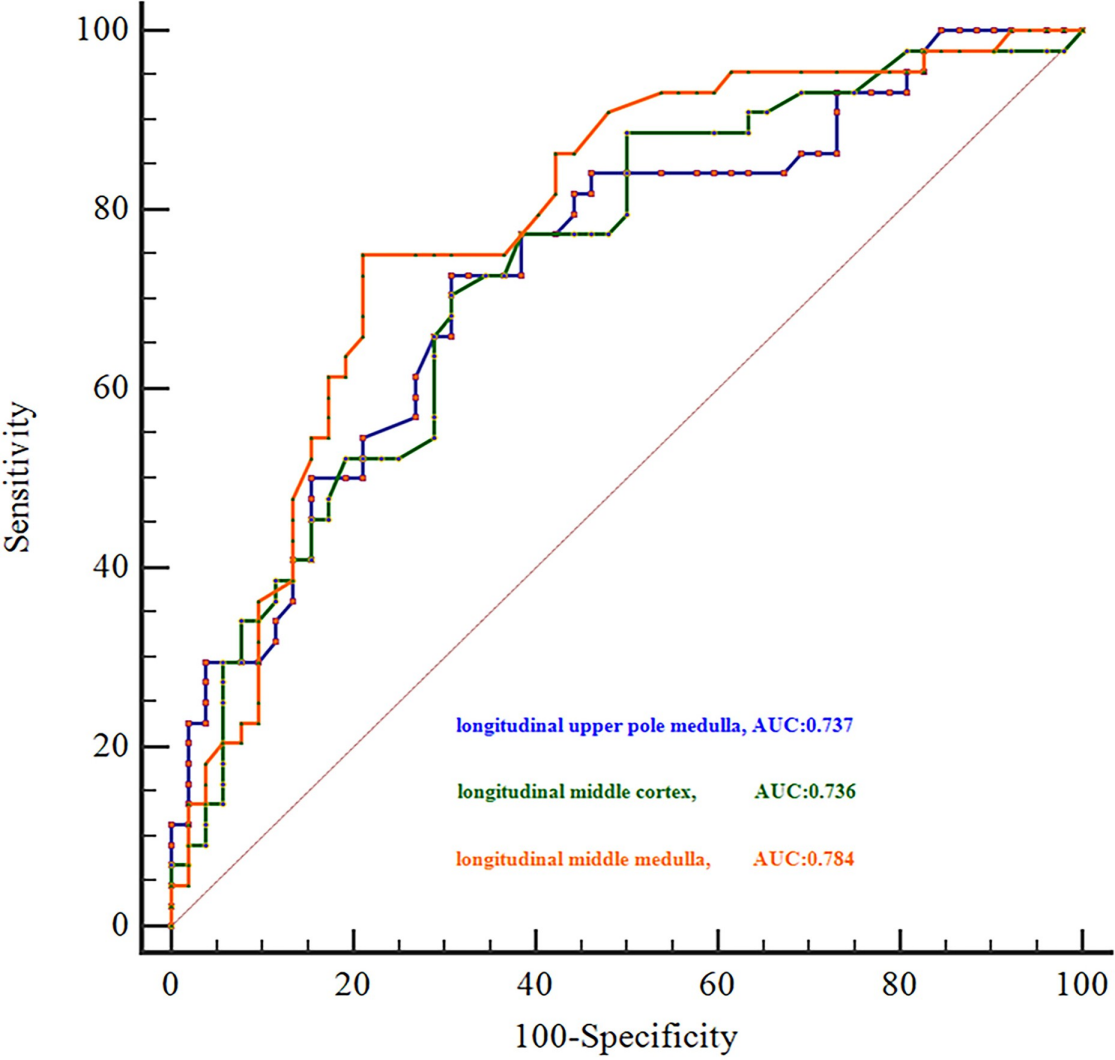
Receiver operating characteristic curves for differentiating between participants with AKI and non-AKI using the 2012 KDIGO criteria as the reference standard. AUC, area under the receiver operating characteristic curve; KDIGO, Kidney Disease: Improving Global Outcomes.

Using SWE measurements, we were able to distinguish participants with AKI from those non-AKI groups based on stiffness values in the upper pole medulla, middle cortex, and middle medulla in the longitudinal plane. The optimal cut-off values for these segments were 9.9 kPa, 2.9 kPa, and 4.4 kPa, respectively. The sensitivity, specificity, positive predictive value, negative predictive value, positive likelihood ratio (LR+), and negative likelihood ratio (LR−) were calculated by cut-off values and are shown in **Table 4**. The area under the ROC curve for differentiating between participants with AKI and those in the non-AKI groups was 0.737 (95% CI: 0.637, 0.822) for the upper pole medulla, 0.736 for middle cortex (95% CI: 0.637, 0.821), and 0.784 for middle medulla (95% CI: 0.688, 0.861) in the longitudinal plane. All these values were statistically significant with *p* < 0.001 **(Fig 3)**.

**Table 4:** Cutoff value, AUC, sensitivity, specificity, positive, and negative likelihood ratios of stiffness values in differentiating participants with AKI and non-AKI by SWE measurements (kPa).

Characteristic	Cutoff value	AUC (95% CI)	Sensitivity(%) (95% CI)	Specificity(%) (95% CI)	Likelihood Ratios (95% CI)	
					Positive	Negative
**Longitudinal plane**						
Upper pole cortex	7.8	0.688[0.586–0.779]	77.27[62.2–88.5]	50.00[35.8–64.2]	1.55[1.1–2.1]	0.45[0.2–0.8]
Upper pole medulla	9.9	0.737[0.637–0.822]	72.73[57.2–85.0]	69.23[54.9–81.3]	2.36[1.5–3.7]	0.39[0.2–0.7]
Middle cortex	2.9	0.736[0.637–0.821]	90.20[78.6–96.7]	50.00[35.8–64.2]	1.80[1.4–2.4]	0.20[0.08–0.5]
Middle medulla	4.4	0.784[0.688–0.861]	74.51[60.4–85.7]	78.85[65.3–88.9]	3.52[2.0–6.1]	0.32[0.2–0.5]
Lower pole cortex	4.1	0.593[0.488–0.692]	56.82[41.0–71.7]	67.31[52.9–79.7]	1.74[1.1–2.8]	0.64[0.4–0.9]
Lower pole medulla	3.9	0.580[0.475–0.680]	52.27[36.7–67.5]	65.38[50.9–78.0]	1.51[0.9–2.4]	0.73[0.5–1.1]
**Transverse plane**						
Upper pole cortex	7.7	0.613[0.508–0.710]	54.55[38.8–69.6]	67.31[52.9–79.7]	1.67[1.0–2.7]	0.68[0.5–1.0]
Upper pole medulla	6.0	0.698[0.595–0.788]	79.55[64.7–90.2]	49.02[34.8–63.4]	1.56[1.1–2.1]	0.42[0.2–0.8]
Middle cortex	7.0	0.592[0.486–0.692]	25.00[13.2–40.3]	94.23[84.1–98.8]	4.33[1.3–14.6]	0.80[0.7–1.0]
Middle medulla	4.1	0.682[0.579–0.774]	68.18[52.4–81.4]	63.46[49.0–76.4]	1.87[1.2–2.8]	0.50[0.3–0.8]
Lower pole cortex	5.5	0.589[0.484–0.688]	27.27[15.0–42.8]	94.23[84.1–98.8]	4.73[1.4–15.7]	0.77[0.6–0.9]
Lower pole medulla	2.1	0.529[0.424–0.633]	86.36[72.6–94.8]	26.92[15.6–41.0]	1.18[1.0–1.4]	0.51[0.2–1.2]

Data are presented as median with interquartile range or frequencies with percentages. SWE, shear wave elastography; 95% CI, 95% confidence interval; AUC, area under the receiver operating characteristic curve.

### Reliability of SWE measurements

The SWE measurements of healthy volunteers and critically ill patients are shown in **S1 File**. The intraobserver reliability of SWE measurement was represented as the ICC in healthy volunteers (ICC: 0.740–0.948) **(S1 Table)**. The interobserver reliability of SWE measurement was represented as the ICC in healthy volunteers (ICC: 0.573–0.870) **(S2 Table)**. The intraobserver reliability of SWE measurement was represented as the ICC in critically ill patients (ICC: 0.801–0.944) **(S3 Table)**. The interobserver reliability of SWE measurement was represented as the ICC in critically ill patients (ICC: 0.521–0.855) **(S4 Table)**.

## Discussion

In our study, we observed that specific kidney regions, particularly the upper pole medulla, middle cortex, and middle medulla in the longitudinal plane, exhibited higher tissue stiffness in AKI patients compared to non-AKI patients. The significant stiffness alterations in these specific regions have the potential to serve as discriminative indicators for differentiating AKI from non-AKI patients. Furthermore, our research elucidates the influence of tissue anisotropy on SWE measurements, emphasizing the observed variability across distinct segments and compartments of the kidney.

AKI has a poor prognosis and can be lethal in critically ill patients. Thus, prevention and early detection of AKI is necessary for timely management. Some new markers, such as TIMP-2 and IGFBP7, are promising and demonstrate good predictive value [22,23]. However, the predictive value of these markers is poor when the timing of renal injury is unknown. Imaging techniques also play a role in the diagnosis of AKI. Charlton et al [24] reported that the noninvasive parameters of CFE-MRI have the potential to distinguish between AKI and CKD following folic acid-induced renal tubular damage. A study [25] demonstrated that the molecular renal probe (MRP) imaging method could detect AKI induced by cisplatin at least 36 hours earlier than other existing noninvasive methods. Compared with the methods mentioned above, SWE is distinct because it relies on biophysical characteristics and can provide quantitative estimates of tissue stiffness. Therefore, this study was conducted to examine the role of kidney tissue stiffness measured by SWE in the diagnosis of AKI.

In our study, we first performed an ICC analysis in healthy volunteers and critically ill patients. Prior studies have demonstrated inconsistent outcomes when assessing the reliability of kidney SWE measurements. Kishimoto et al [26] reported that the inter- and intraoperator ICC were 0.75 or higher in healthy volunteers. Another study [27] reported good interoperator agreement in the right kidney (ICC = 0.71). Conversely, Sorana D et al [19] emphasized the significant variability in intra-examination stiffness, with a coefficient of variation from 2.21% to 45.04%. Similarly, inter-examination stiffness exhibited heterogeneity, with values between 28.66% and 42.38%. However, our analysis revealed that the SWE measurements in the two cohorts both indicated fair to good or even excellent inter- and intraobserver reliability, although repeated measurements were taken for different segments and compartments of kidney tissue in the longitudinal and transverse planes. In our present study, we noted a good reproducibility of SWE measurements in the kidney; this might be ascribed to the fact that participants were instructed to remain motionless during measurement. Additionally, our ultrasound machines were set to image quality mode, and the IQR was < 30% of the median value. These methods may have minimized the variability of the SWE measurements.

The kidney is highly heterogeneous and anisotropic. Moreover, the intrinsic structure of the parenchyma is highly oriented. According to previous studies [17,18,28], by using SWE to measure stiffness values in the kidney, the effect of anisotropy can affect the propagation speed of shear waves, which lead to inconsistent SWE values in the cortex and medulla in both the axial and transverse planes. Our results showed that, with the exception of the segments of upper pole cortex, medulla in both the longitudinal and transverse planes showed significant differences, the stiffness values did not differ between the longitudinal and transverse planes among the other segments and compartments. These findings are contradictory to previously reported results. We considered that the upper pole of the right kidney lies medially and posteriorly, and its position is oblique and further away from the probe, making it difficult for the ultrasound beam to align the shear waves with the axis of the pyramid in either a parallel or perpendicular direction. Moreover, it is unlikely that all renal pyramids aligned either parallel or perpendicular to the transducer. Therefore, the shear wave may be tilted by some renal pyramids. The shear wave which is oblique to the renal pyramid may be responsible for the uneven results. Our results suggest that the transducer-to-kidney angle may affect measurements of tissue stiffness. Therefore, it is imperative that the ultrasonic transducer is properly positioned to obtain consistent measurements.

Previous study [29] had found that the YM gradually increased from non-AKI patients to patients with AKI III in the upper and middle poles cortex in the longitudinal plane. In our expanded study, we investigated the role of SWE as a complementary diagnostic tool for AKI. We found that patients in the AKI group exhibited significantly higher stiffness values in specific kidney regions compared to the non-AKI group. The increased tissue stiffness observed within specific kidney regions can be attributed to an array of underlying pathological changes linked to AKI. Contributing factors such as cellular swelling [30], vascular pressure [17], varying blood flow perfusion patterns [31], and region-specific vulnerability [32] are posited as probable drivers of this increased stiffness. Liu et al. [31] have elucidated the crucial role of renal perfusion in determining elasticity. Their research showed a connection between extended renal vein ligation and increased YM values in various kidney regions. Variations in kidney blood flow and intra-renal pressure, often observed in AKI, remain areas that warrant further investigation. Currently, the prognosis and management of AKI predominantly rely on sCr levels and urine output. In our study, we introduced SWE measurements as a novel method to differentiate between AKI and non-AKI groups. The results corroborated the notion that alterations in kidney tissue stiffness can serve as indicators for AKI detection.

Our study is subject to several limitations that warrant careful consideration. First, we acknowledge the limitations of our single-center study design and the relatively small cohort of patients with AKI. This could potentially limit the generalizability of our findings to broader patient populations and stratified analyses based on certain AKI causes. To address this, future research could involve multi-center collaborations to enhance the diversity and representativeness of the study cohort. Second, the absence of analyses related to fluid management and kidney blood flow or perfusion is noteworthy. Incorporating these factors into future investigations could provide a more comprehensive understanding of the complex interplay between kidney function, hemodynamics, and SWE measurements. Third, the lack of analysis concerning the correlation between kidney stiffness values and AKI grades is another limitation. Future research endeavors could explore this correlation to establish potential associations between SWE measurements and the severity of AKI, which could inform risk stratification and clinical decision-making. Lastly, it is important to note that the limited sample size in our dataset may have reduced the statistical power to detect subtle sex or gender-related variations in SWE measurements among AKI patients. Therefore, future studies with larger and more diverse cohorts are needed to thoroughly investigate the influence of gender on SWE measurements in AKI patients, potentially informing personalized approaches to patient management.

## Conclusions

In conclusion, SWE can serve as a reliable technique for detecting abnormal stiffness value changes in AKI among critically ill patients, and this method may confer advantages in clinical practice. Further studies with larger study populations are needed to verify these results and determine the threshold for detecting AKI using SWE.

## Supporting information

S1 Table Intraobserver reliability of stiffness value by SWE measurements in different segments and compartments of kidney in healthy volunteers (kPa). (DOCX)

S2 Table Interobserver reliability of stiffness value by SWE measurements in different segments and compartments of kidney in healthy volunteers (kPa). (DOCX)

S3 Table Intraobserver reliability of stiffness value by SWE measurements in different segments and compartments of kidney in critically ill patients (kPa). (DOCX)

S4 Table Interobserver reliability of stiffness value by SWE measurements in different segments and compartments of kidney in critically ill patients (kPa). (DOCX)

S1 File Reliability of SWE measurements in healthy volunteers and critically ill patients. (DOCX)

S2 File STROBE-checklist. (DOCX)
