# Supplementary material for: Shear wave elastography: A noninvasive approach for assessing acute kidney injury in critically ill patients
Source: PLoS One. 2024 Jan 11;19(1):e0296411. doi: 10.1371/journal.pone.0296411 (PMC10783713; doi:10.1371/journal.pone.0296411)
Supplement: S1 Table — (DOCX) [file pone.0296411.s001.docx]

| **S1 Table. Intraobserver reliability of stiffness value by SWE measurements in different segments and compartments of kidney in healthy volunteers (kPa)** | | | | | |
| --- | --- | --- | --- | --- | --- |
| **Characteristic** | **Operator A** | **Operator A'** | **ICC** | **95% CI** | ***p*-value** |
| Longitudinal upper pole cortex | 12.70 (10.3–14.30) | 11.10 (8.55–12.35) | 0.740 | 0.217, 0.903 | ＜0.001 |
| Longitudinal upper pole medulla | 12.10 (10.2–13.05) | 11.50 (10.0–13.10) | 0.833 | 0.658, 0.923 | ＜0.001 |
| Longitudinal middle cortex | 4.00 (3.30–4.80) | 3.90 (2.75–4.55) | 0.842 | 0.677, 0.927 | ＜0.001 |
| Longitudinal middle medulla | 5.00 (3.30–5.70) | 4.70 (3.50–5.90) | 0.939 | 0.867, 0.973 | ＜0.001 |
| Longitudinal lower pole cortex | 3.50 (2.90–4.80) | 3.60 (2.25–5.05) | 0.873 | 0.735, 0.941 | ＜0.001 |
| Longitudinal lower pole medulla | 4.80 (3.10–5.85) | 4.20 (3.10–5.65) | 0.819 | 0.629, 0917 | ＜0.001 |
| Transverse upper pole cortex | 6.60 (4.65–8.25) | 6.80 (4.55–8.05) | 0.863 | 0.714, 0.937 | ＜0.001 |
| Transverse upper pole medulla | 7.10 (5.30–10.55) | 8.20 (4.90–11.30) | 0.948 | 0.865, 0979 | ＜0.001 |
| Transverse middle cortex | 4.60 (3.90–5.70) | 4.80 (3.35–6.00) | 0.875 | 0.739, 0.942 | ＜0.001 |
| Transverse middle medulla | 6.00 (3.70–7.90) | 5.10 (3.25–8.00) | 0.865 | 0.715, 0.937 | ＜0.001 |
| Transverse lower pole cortex | 3.80 (2.50–5.40) | 3.80 (3.00–5.25) | 0.836 | 0.664, 0.924 | ＜0.001 |
| Transverse lower pole medulla | 5.10 (3.50–6.75) | 3.80 (2.95–6.40) | 0.838 | 0.664, 0.926 | ＜0.001 |
| Data are presented as median with interquartile range. Operator A: B-H Q; Operator A': B-H Q; measurement one day interval; SWE: shear wave elastography; ICC: intraclass correlation coefficient; 95% CI: 95% confidence interval | | | | | |
