# Supplementary material for: Shear wave elastography: A noninvasive approach for assessing acute kidney injury in critically ill patients
Source: PLoS One. 2024 Jan 11;19(1):e0296411. doi: 10.1371/journal.pone.0296411 (PMC10783713; doi:10.1371/journal.pone.0296411)
Supplement: S2 Table — (DOCX) [file pone.0296411.s002.docx]

| **S2 Table. Interobserver reliability of stiffness value by SWE measurements in different segments and compartments of kidney in healthy volunteers (kPa)** | | | | | |  |
| --- | --- | --- | --- | --- | --- | --- |
| **Characteristic** | **Operator A** | **Operator B** | **ICC** | **95%CI** | ***p*-value** | |
| Longitudinal upper pole cortex | 12.70 (10.35–14.30) | 10.00(9.05–11.70) | 0.640 | 0.090, 0.857 | ＜0.001 | |
| Longitudinal upper pole medulla | 12.10 (10.25–13.05) | 10.70(10.10–13.45) | 0.707 | 0.441, 0.859 | ＜0.001 | |
| Longitudinal middle cortex | 4.00 (3.30–4.80) | 4.40 (3.20–5.20) | 0.815 | 0.628, 0.913 | ＜0.001 | |
| Longitudinal middle medulla | 5.00 (3.30–5.70) | 4.90 (3.55–7.65) | 0.870 | 0.713, 0.942 | ＜0.001 | |
| Longitudinal lower pole cortex | 3.50 (2.90–4.80) | 2.90 (2.30–5.75) | 0.829 | 0.649, 0.921 | ＜0.001 | |
| Longitudinal lower pole medulla | 4.80 (3.10–5.85) | 4.20 (2.95–5.90) | 0.759 | 0.526, 0886 | ＜0.001 | |
| Transverse upper pole cortex | 6.60 (4.65–8.25) | 6.30 (4.40–8.80) | 0.573 | 0.238, 0.786 | 0.001 | |
| Transverse upper pole medulla | 7.10 (5.30–10.55) | 9.20 (6.60–11.60) | 0.766 | 0.481, 0.896 | ＜0.001 | |
| Transverse middle cortex | 4.60 (3.90–5.70) | 4.40 (2.75–6.45) | 0.836 | 0.663, 0925 | ＜0.001 | |
| Transverse middle medulla | 6.00 (3.70–7.90) | 6.00 (3.20–8.45) | 0.813 | 0.625, 0913 | ＜0.001 | |
| Transverse lower pole cortex | 3.80 (2.50–5.40) | 3.50 (2.25–5.60) | 0.852 | 0.696, 0.931 | ＜0.001 | |
| Transverse lower pole medulla | 5.10 (3.50–6.75) | 4.50 (3.30–6.05) | 0.802 | 0.605, 0.907 | ＜0.001 | |
| Data are presented as median with interquartile range. Operator A: B-H Q; Operator B: C-Y S; SWE: shave wave elastography; ICC: intraclass correlation coefficient; 95% CI: 95% confidence interval | | | | | |  |
