# Supplementary material for: Shear wave elastography: A noninvasive approach for assessing acute kidney injury in critically ill patients
Source: PLoS One. 2024 Jan 11;19(1):e0296411. doi: 10.1371/journal.pone.0296411 (PMC10783713; doi:10.1371/journal.pone.0296411)
Supplement: S3 Table — (DOCX) [file pone.0296411.s003.docx]

| **S3 Table. Intraobserver reliability of stiffness value by SWE measurements in different segments and compartments of kidney in critically ill patients (kPa)** | | | | | |
| --- | --- | --- | --- | --- | --- |
| **Characteristic** | **Operator A** | **Operator A'** | **ICC** | **95% CI** | ***p*-value** |
| Longitudinal upper pole cortex | 10.40 (4.80–14.33) | 8.50 (5.83–11.48) | 0.882 | 0.703, 0.951 | ＜0.001 |
| Longitudinal upper pole medulla | 8.90 (5.25–13.68) | 8.30 (5.13–12.10) | 0.920 | 0.781, 0968 | ＜0.001 |
| Longitudinal middle cortex | 4.85 (2.40–7.15) | 5.15 (2.80–8.00) | 0.961 | 0.913, 0.983 | ＜0.001 |
| Longitudinal middle medulla | 3.55 (2.33–6.70) | 3.50 (2.35–6.95) | 0.920 | 0.824, 0.965 | ＜0.001 |
| Longitudinal lower pole cortex | 3.35 (2.23–5.55) | 3.10 (1.90–5.20) | 0.801 | 0.567, 0913 | ＜0.001 |
| Longitudinal lower pole medulla | 2.95 (2.20–4.88) | 2.65 (2.10–5.18) | 0.835 | 0.658, 0.925 | ＜0.001 |
| Transverse upper pole cortex | 8.00 (4.00–11.10) | 8.55 (3.65–12.88) | 0.832 | 0.644, 0.925 | ＜0.001 |
| Transverse upper pole medulla | 7.40 (3.85–12.05) | 6.90 (2.98–10.35) | 0.932 | 0.850, 0.970 | ＜0.001 |
| Transverse middle cortex | 5.10 (2.55–6.73) | 4.80 (2.80–6.38) | 0.944 | 0.876, 0.975 | ＜0.001 |
| Transverse middle medulla | 3.95 (2.23–6.78) | 3.20 (1.90–5.20) | 0.869 | 0.718, 0.942 | ＜0.001 |
| Transverse lower pole cortex | 3.00 (1.80–4.40) | 3.20 (2.05–4.05) | 0.804 | 0.587, 0.913 | ＜0.001 |
| Transverse lower pole medulla | 3.05 (1.98–3.95) | 3.35 (2.18–4.73) | 0.934 | 0.851, 0.971 | ＜0.001 |
| Data are presented as median with interquartile range. Operator A: B-H Q; Operator A': B-H Q, measurement one day interval; SWE: shear wave elastography; ICC: intraclass correlation coefficient; 95% CI: 95% confidence interval | | | | | |
