# Supplementary material for: Shear wave elastography: A noninvasive approach for assessing acute kidney injury in critically ill patients
Source: PLoS One. 2024 Jan 11;19(1):e0296411. doi: 10.1371/journal.pone.0296411 (PMC10783713; doi:10.1371/journal.pone.0296411)
Supplement: S4 Table — (DOCX) [file pone.0296411.s004.docx]

| **S4 Table. Interobserver reliability of stiffness value by SWE measurements in different segments and compartments of kidney in critically ill patients (kPa)** | | | | | |
| --- | --- | --- | --- | --- | --- |
| **Characteristic** | **Operator A** | **Operator B** | **ICC** | **95% CI** | ***p*-value** |
| Longitudinal upper pole cortex | 10.40 (4.80–14.33) | 11.45 (5.88–14.48) | 0.604 | 0.271, 0.808 | 0.001 |
| Longitudinal upper pole medulla | 8.90 (5.25–13.68) | 9.25 (4.85–11.85) | 0.596 | 0.261, 0.803 | 0.001 |
| Longitudinal middle cortex | 4.85 (2.40–7.15) | 6.75 (3.18–8.85) | 0.855 | 0.698, 0.934 | ＜0.001 |
| Longitudinal middle medulla | 3.55 (2.33–6.70) | 4.95 (2.32–6.30) | 0.648 | 0.342, 0.830 | ＜0.001 |
| Longitudinal lower pole cortex | 3.35 (2.23–5.55) | 2.80 (2.10–4.50) | 0.792 | 0.565, 0.907 | ＜0.001 |
| Longitudinal lower pole medulla | 2.95 (2.20–4.88) | 3.15 (2.23–4.70) | 0.722 | 0.461, 0.869 | ＜0.001 |
| Transverse upper pole cortex | 8.00 (4.00–11.10) | 5.45 (2.40–8.55) | 0.527 | 0.152, 0.769 | 0.005 |
| Transverse upper pole medulla | 7.40 (3.85–12.05) | 6.90 (4.03–10.55) | 0.686 | 0.404, 0.850 | ＜0.001 |
| Transverse middle cortex | 5.10 (2.55–6.73) | 4.55 (2.28–6.75) | 0.699 | 0.417, 0.858 | ＜0.001 |
| Transverse middle medulla | 3.95 (2.23–6.78) | 4.35 (2.23–7.90) | 0.717 | 0.453, 0.867 | ＜0.001 |
| Transverse lower pole cortex | 3.00 (1.80–4.40) | 3.05 (2.13–5.28) | 0.525 | 0.153, 0767 | 0.005 |
| Transverse lower pole medulla | 3.05 (1.98–3.95) | 6.25 (1.78–6.00) | 0.678 | 0.385, 0.846 | ＜0.001 |
| Data are presented as median with interquartile range. Operator A: B-H Q; Operator B: C-Y S; SWE: shear wave elastography; ICC: intraclass correlation coefficient; 95% CI: 95% confidence interval | | | | | |
