# Supplementary material for: Shear wave elastography: A noninvasive approach for assessing acute kidney injury in critically ill patients
Source: PLoS One. 2024 Jan 11;19(1):e0296411. doi: 10.1371/journal.pone.0296411 (PMC10783713; doi:10.1371/journal.pone.0296411)
Supplement: S1 File — (DOCX) [file pone.0296411.s005.docx]

**Reliability of SWE measurements in healthy volunteers and critically ill patients**

**Inclusion and exclusion criteria**

Twenty-four critically ill patients were enrolled for a shear wave elastography (SWE) examination. The inclusion and exclusion criteria pertaining to the selection of critically ill patients for this study are detailed comprehensively in the main manuscript (see Page 5, under the 'Participants' section). Another 25 healthy volunteers were screened for the absence of common diseases, including CKD (and/or an estimated glomerular filtration rate (eGFR) < 60 mL/min), hypertension, diabetes, and cardiovascular disease. None of the participants had abnormal kidney function laboratory tests or abnormal kidney imaging findings, such as cysts or hydronephrosis, on conventional B-mode US. The inclusion criteria for healthy volunteers included (a) age greater than 18 years, (b) BMI< 35 kg/m^2^, (c) not pregnant or nursing, and (d) structurally normal kidneys on conventional B-mode US.

**SWE Protocol**

When investigating the interobserver reliability, SWE measurements were initially conducted by the first sonographer (B.H) and subsequently repeated by a second sonographer (C.Y.) on the same day. Both sonographers had prior training in SWE and were blind to the participants' clinical and laboratory data to ensure unbiased measurements. In examining intraobserver reliability, the first sonographer performed SWE measurements on each participant consistently, with sessions spaced at one-day intervals. The two sonographers were completely blinded to each other's SWE imaging findings. The obtained data were used for the analysis of intra- and interobserver reliability of SWE measurements.

**Results**

First, 25 healthy volunteers were enrolled. The median age was 34 years (IQR: 28–58 years), the median BMI was 21.63 kg/m^2^ (IQR: 19.98–23.85 kg/m^2^), and 17 (68%) participants were men. The stiffness values of the cortex and medulla in different segments and compartments of the kidney between the longitudinal and transverse planes were similar between sexes (*p*＞0.05). The kidney length and RI were greater in men than in women (*p*＜0.05). The intraobserver reliability, measured in the longitudinal upper pole medulla, middle cortex, middle medulla, lower pole cortex, and lower pole medulla, was excellent (ICC: 0.833–0.939), and that measured in the transverse upper pole cortex, upper pole medulla, middle cortex, middle medulla, lower pole cortex, and lower pole medulla was also excellent (ICC: 0.836–0.948). However, the intraobserver reliability for the longitudinal upper pole cortex was fair to good (ICC: 0.740). The interobserver reliability, measured in the longitudinal middle cortex, middle medulla, lower pole cortex, and lower pole medulla, was excellent (ICC: 0.759–0.870), and that measured in the transverse upper pole medulla, middle cortex, middle medulla, lower pole cortex, and lower pole medulla was also excellent (ICC: 0.766-0.852). However, the interobserver reliability for the longitudinal upper pole cortex, upper pole medulla, and the transverse upper pole cortex were fair to good (ICC: 0.573-0.707). We then enrolled 24 critically ill patients. The median age was 59 years (IQR: 54–75 years). The median BMI was 23.67 kg/m^2^ (IQR: 21.16–27.11 kg/m^2^), and 17 (67%) participants were men. The stiffness values of the cortex and medulla in different segments and compartments of the kidney between the longitudinal and transverse planes were similar between sexes (*p*＞0.05). The intraobserver reliability, measured in the longitudinal upper pole cortex, upper pole medulla, middle cortex, middle medulla, lower pole cortex, and lower pole medulla, was excellent (ICC: 0.801–0.920), and that measured in the transverse upper pole cortex, upper pole medulla, middle cortex, middle medulla, lower pole cortex, and lower pole medulla was also excellent (ICC: 0.804–0.944). The interobserver reliability measured in the longitudinal middle cortex and lower pole cortex was excellent (ICC: 0.792–0.855), whereas that measured in the transverse kidney upper pole cortex, upper pole medulla, middle cortex, middle medulla, lower pole cortex, lower pole medulla, longitudinal kidney upper pole cortex, upper pole medulla, middle medulla, and lower pole medulla was fair to good (ICC: 0.521–0.722).
